# Psychometric testing of the support and control in birth scale

**DOI:** 10.1186/s12884-020-02888-x

**Published:** 2020-05-14

**Authors:** Shu-Yu Liu, Yu-Ying Lu, Meei-Ling Gau, Chieh-Yu Liu

**Affiliations:** 1 National Taipei University of Nursing and Health Sciences, No. 365, Mingde Rd., Beitou Dist., Taipei City, 112 Taiwan, Republic of China; 2 Lo-Hsu Medical Lotung poh-Ai Hospital, No. 83. Nanchang St., Luodong Township, Yilan County, 265 Taiwan, Republic of China; 3 School of Nursing, National Taipei University of Nursing and Health Sciences, No. 365, Mingde Rd., Beitou Dist., Taipei City, 112 Taiwan, Republic of China; 4 Department of Nurse-Midwifery and Women Health; Research Center for Healthcare Industry Innovation, National Taipei University of Nursing and Health Sciences, No. 365, Mingde Rd., Beitou Dist., Taipei City, 112 Taiwan, Republic of China; 5 Department of Speech Language Pathology and Audiology, National Taipei University of Nursing and Health Sciences, No. 365, Mingde Rd., Beitou Dist, Taipei City, 112 Taiwan, Republic of China

**Keywords:** Labor support, Chinese version of support and control in birth (C-SCIB) scale, Reliability and validity test, Confirmatory factor analysis

## Abstract

**Background:**

The Support and Control in Birth (SCIB) scale primarily measures the perceived support and control of expectant mothers during childbirth, thereby obtaining an understanding of their birth experiences. The advantages of this scale are its good reliability and validity and that it consolidates birth support and control. However, a Chinese version of the scale has yet to be developed. Therefore, this study sought to evaluate the validity and reliability of a Chinese version of the Support and Control in Birth Scale (C-SCIB).

**Methods:**

A total of 228 postpartum women participated in this study. The C-SCIB scale was developed through a translation and back translation, followed by an evaluation of its content validity by a group of experts. Cronbach’s α internal consistency and test-retest reliability were used to test the reliability of the scale. In addition, criterion-related validity (predictive validity and concurrent validity) and construct validity were used to test the validity of the scale.

**Results:**

The C-SCIB scale showed good results in terms of the item-level and scale-level content validity indices. The Cronbach’s α internal consistency was 0.81, and its test-retest reliability was 0.96. The confirmatory factor analysis results showed the overall goodness-of-fit was parsimony fit indices. The predictive validity analysis showed a significant positive correlation between the C-SCIB scale and the Questionnaire Measuring Attitudes About Labor and Delivery (*r* = 0.31, *p* < 0.01). Furthermore, the concurrent validity analysis showed a significant and moderate correlation between the C-SCIB and the Bryanton Adaptation of the Nursing Support in Labor Questionnaire (*r* = 0.49, *p* < 0.01) as well as the Labor Agentry Scale (*r* = 0.51, *p* < 0.01).

**Conclusion:**

The C-SCIB scale was proven to have good reliability and validity, and thus can be used to measure the degree of support and the locus of control perceived by expectant women during labor.

## Background

A successful birth experience rewards a woman with a sense of accomplishment, allows her to develop a better self-concept, and assists her in her future role as a mother [1, 2]. A mother’s birth experience is governed by various factors, such as the degree of pain, control, support, and obstetrical interventions [3]. During the process of childbirth, being capable of making decisions, having a sense of control over one’s body, having good communication with nurses, and adequate pain relief allow a woman to have a higher degree of birth satisfaction. There are two main factors affecting such satisfaction; one is support during childbirth, and the other is the woman’s sense of control over her body during childbirth [4].

An internal locus of control is the most important basis for the satisfaction of women during childbirth. An expectant woman’s internal locus of control over time and space indirectly affects her self-esteem. Hence, if she loses her internal locus of control, she would also lose her self-esteem, which would then generate negative emotions and negative self-concepts [5]. Schroeder divided a woman’s locus of control during childbirth into three aspects: control of pain, emotions, and interpersonal relationships. The author suggested that control is an important factor affecting birth satisfaction and experience. A woman’s birth satisfaction and experience is lowered if her locus of control over her birth anticipation and her experience becomes inconsistent [6]. Studies have pointed out a significant association between a woman’s sense of control and pain relief during childbirth [7, 8]. Hence, maintaining a sense of control during childbirth is vital for expectant women.

In addition, Field [9] mentioned that nursing support is another important indicator affecting an expectant woman’s birth experience. The continuous support and various non-pharmacological pain relief measures provided by nurses to an expectant woman can increase her adaptability and generate a positive and satisfying birth experience [10, 11]. According to Hodnett [11], nursing support increases the participation of an expectant mother during her process of childbirth, allowing her to be in control of her own state and make decisions, thereby maintaining her locus of control. The results of a local study investigating the correlation between labor support and labor control with a woman’s birth satisfaction showed that a significant correlation exists between labor support, labor control, and birth satisfaction. The women in that study perceived that the emotional support provided by nurses was beneficial for alleviating their labor pains, and the accompanying nurses gave them a better sense of control during childbirth [12]. Therefore, nursing support assists a woman in obtaining a locus of control during childbirth and has a positive influence on her birth experience.

In a Swedish study, 2541 women were surveyed and four risk factors of a negative birth experience were delineated: (1) Unexpected medical problems, such as emergency operative delivery, augmentation, and prolonged labor; (2) A woman’s social life, such as unintended pregnancy and a lack of partner support; (3) A woman’s feelings during labor, such as pain and a lack of a sense of control; and (4) The caregivers’ influences, such as inadequate prenatal preparation, lack of labor support, and anesthetic pain management. The authors concluded that the lack of a sense of control and the lack of support from medical staff are the most important risk factors [13].

Another study pointed out that the pain experienced during childbirth, degree of social support, self-efficacy, self-control, internal locus of control, anxiety, and coping with stress are also important factors affecting a negative birth experience [14]. Tokiwa [15] studied the correlation between a woman’s early postpartum depression and her birth experience and suggested that a woman with a lower level of birth satisfaction has a higher possibility of developing postpartum depression. A study by Creedy, Shochet and Horsfall [16] revealed that negative birth experiences were prevalent in women who experienced a high degree of medical intervention during childbirth (such as emergency operative delivery) and in those unsatisfied during childbirth and lacking adequate care. These women were susceptible to developing traumatic symptoms. Bruno et al. [17] indicated that negative emotional experiences and expressions during childbirth can be considered a vulnerability factor for the onset of postpartum emotional disorders. In particular, the expression of negative emotions to other people or objects in the environment heralds the onset of maternity blues. Another study revealed a significant effect of maternal perceived social support on postpartum depression [18].

In short, a woman’s birth experience has a profound influence on her, and nursing support and control play a vital role in a woman’s birth experience. Nursing support assists an expectant mother in maintaining her locus of control, while a woman’s sense of control is the most essential factor governing her birth experience [5].

In 2009, Ford et al. [19] developed the Support and Control in Birth (SCIB) scale, which primarily measures the feelings perceived by expectant women towards the support and control during their process of childbirth. This scale offers good reliability and validity, in addition to consolidating labor support and control. The scale can be used in studies involving issues of labor control and support.

### Aim of the study

The aim of this study was to develop a Chinese version of the SCIB (C-SCIB) scale and then test the reliability and validity of the scale.

## Methods

### Setting and participants

Convenience sampling was adopted in this study. The participants consisted of postpartum women. This study took place in a regional teaching hospital in Taiwan. The inclusion criteria were as follows:

1. One-week postpartum women with no complications during pregnancy, labor, and delivery.
2. Women who were pregnant for at least 37 weeks; had a natural vaginal delivery with a duration of labor of over 3 h; and for whom the Apgar Score of the newborn was above 7 and taken within 1 to 5 min post-childbirth with no abnormalities or complications observed.
3. Expectant Taiwanese women who can understand, speak, read, and write Mandarin Chinese.

Women with a history of depression, postpartum depression, or the use of antidepressants were excluded. A total of 228 postpartum women participated in this study**.** This study was conducted after gaining approval from an Institutional Review Board (IRB). Subjects who met the inclusion criteria were briefed on the study proposal and aim by the researcher. Those who agreed to participate in this study had to fill out a consent form first, followed by a questionnaire. For women under 18 years of age, we obtained the consent and contact details of participants’ guardian prior to enrolment of the participants.

### Procedure

**Fig. 1:**
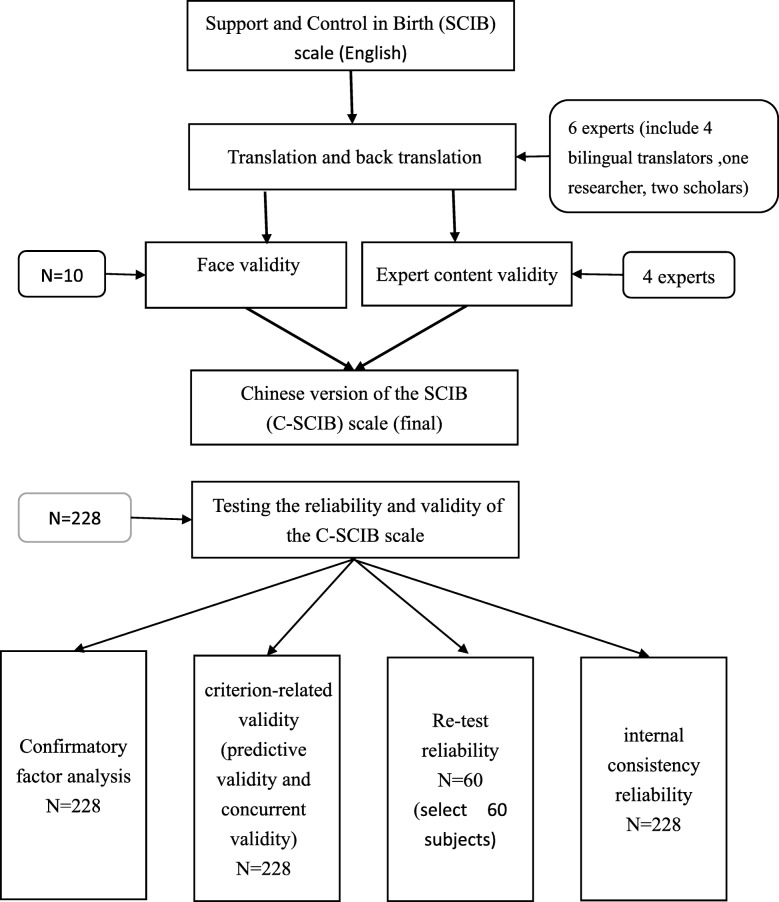
The procedure of Chinese version of the SCIB (C-SCIB) scale

The main objective of this study was to develop a Chinese version of the SCIB (C-SCIB) scale and test its reliability and validity in clinical applications. The researcher of this study followed the guidelines developed by Beaton et al. [20] for cross-cultural translations of scales. First, the SCIB scale was translated and back translated into Mandarin Chinese, and then the reliability and validity of the C-SCIB scale were tested (Fig. 1).

Translation and back translation: The translation process consisted of the following steps:

- Step 1: Two bilingual translators translated the original (English) SCIB scale into Mandarin Chinese.
- Step 2: The researcher and a scholar discussed each question in both translations and reached a consensus on the C-SCIB scale.
- Step 3: Another pair of bilingual translators then translated the C-SCIB scale back into English. The researcher and a different scholar discussed and compared the accuracy of the back translation in expressing the meanings of the original SCIB scale. This step was completed when the C-SCIB scale and the SCIB scale were equivalent.
- Step 4: The expert validity of the C-SCIB scale was tested by enlisting four experts to evaluate the meaning, content applicability, and clarity of the translation. This was followed by the completion of the draft C-SCIB scale.
- Step 5: The researcher selected 10 postpartum women from the postpartum ward that met the inclusion criteria of this study, so as to test the difficulty of the meanings of the C-SCIB scale and the estimated time of scale administration (that is, to test the face validity of the scale). The selected participants were also asked if they encountered any difficulties in understanding or if the meanings of the scale were unclear. Then, the final version of the C-SCIB scale was completed.

### Testing the reliability and validity of the C-SCIB scale

The reliability and validity of the C-SCIB scale’s final version had to be tested as well. In terms of reliability, internal consistency reliability (Cronbach’s alpha) and test-retest reliability (60 participants were selected 2 weeks later to fill out the scale again) were used in this study. According to Gray, Grove, and Sutherland [21], the main assumption of test–retest reliability is that the time period between tests will be long enough to prevent learning, carry-over effects, or recall. To meet the time period condition, the test and retest administration were separated by a two-week time interval. In addition, a comparison of the demographic characteristics of the 60 participants selected for the retest and the total sample of 228 participants was conducted to ensure the homogeneity of the samples.

Face validity and expert validity were used to test the content validity. Ten postpartum women were asked to test the face validity. The expert validity of the scale was assessed by four experts (two obstetricians, a clinical expert, and a professor). Confirmatory factor analysis was used to test the fit of the structural model. The criterion-related validity (predictive validity and concurrent validity) of the C-SCIB scale was tested as well.

### Statistical analysis

SPSS (21.0) statistical software and LISREL (8.54) structural equation modeling software were used for data treatment and analysis. SPSS 21.0 was used in this study to check the basic demographic data and the obstetrical data of the participants and the descriptive statistics data, as well as to perform reliability analysis and criterion-related validity.

In terms of descriptive statistics, the distributions of the demographic data and the obstetrical data were expressed in percentages, means, standard deviations, and frequency distributions. Cronbach’s α was used to test the internal consistency reliability. The test-retest reliability was evaluated 2 weeks after the initial administration of the scale. The sample consisted of 60 participants, and Pearson’s correlation was used for analysis.

In terms of expert content validity, a content validity index (CVI) was used, which consisted of an item-level CVI (I-CVI) and a scale-level CVI (S-CVI). The Labor Agentry Scale (LAS) and the Bryanton Adaptation of the Nursing Support in Labor Questionnaire (BANSILQ) were used to test the concurrent validity of the C-SCIB scale, whereas a birth experience scale (Questionnaire Measuring Attitudes About Labor and Delivery Experience, QMAALD) was used to test the predictive validity of the C-SCIB scale. Pearson’s correlation was used for testing. For construct validity, LISREL (8.54) structural modeling software was used for confirmatory factor analysis. Structural models were drawn using LISREL (8.54), so as to assess if the fit of the theoretical model was in line with the overall goodness of fit.

## Results

### Demographic characteristics of participants

When conducting the research, the researcher administered the C-SCIB scale to 252 one-week postpartum women. After omitting 24 invalid responses, there were 228 valid responses (90.4%) left. Of the 228 participants with valid responses, the first 60 women who completed the C-SCIB were selected to evaluate the scale’s test-retest reliability. The age of the 228 participants ranged from 16 to 46 years, with a mean of 28.99 ± 5.171 years. Most of the participants had a college degree (*n* = 69, 30.3%); 146 participants had participated in prenatal education (64.0%). Most of the participants were accompanied by their husbands during their labor and delivery (*n* = 205, 89.9%). Most of the participants were having their first baby (*n* = 132, 55.0%) (Table 1).

**Table 1:** Characteristics of the study participants (*N* = 228)

Variable	n	%
Age *(M ± SD)*	28.99 ± 5.171	
Education level		
Less than a vocational school diploma	26	11.4
Vocational school diploma	61	26.8
Junior college	64	28.1
College degree	69	30.3
Doctoral degree	8	3.5
Participation in prenatal education		
Yes	146	64.0
No	82	36.0
Main companion during labor (multiple choice)^a^		
None	3	1.3
Husband	205	89.9
Mother	47	20.6
Mother-in-law	27	11.8
Other family members	20	8.8
Friend	3	1.3
Main companion during delivery		
None	52	22.8
Husband	166	72.8
Mother	18	7.9
Mother-in-law	9	3.9
Other family members	7	3.1
Friend	3	1.3
Gestational weeks (*M ± SD*)	ˇ 38.86 ± 1.080	
Parity		
First	122	53.5
Second	84	36.8
Third	16	7.0
Fourth	3	1.3
Fifth and above	3	1.3

^a^ Multiple choice questions are counted by head

The demographic characteristics of the 60 participants selected for the retest are shown in Table 2. There were no statistically significant differences in demographic characteristics between the participants selected for the retest and the total sample of 228 participants (*p* > 0.05).

**Table 2:** Characteristics of the re-tested participants drawn from the overall study sample of 228 participants (*N* = 60)

Variable	n	%
Age *(M ± SD)*	29.28 ± 4.875	
Education level		
Less than a vocational school diploma	8	13.3
Vocational school diploma	14	23.3
Junior college	17	28.3
College degree	19	31.7
Doctoral degree	2	3.3
Participation in prenatal education		
Yes	43	71.7
No	17	28.3
Main companion during labor (multiple choice)^a^		
None	1	1.7
Husband	54	90.0
Mother	15	25.0
Mother-in-law	10	16.7
Other family members	5	8.3
Friend	0	0
Main companion during delivery		
None	8	13.3
Husband	47	78.3
Mother	8	13.3
Mother-in-law	6	10.0
Other family members	5	8.3
Friend	1	1.7
Gestational weeks (*M ± SD*)	ˇ 38.92 ± 0.996	
Parity		
First	34	56.7
Second	18	30.0
Third	6	10.0
Fourth	1	1.7
Fifth and above	1	1.7

^a ^Multiple choice questions are counted by head

### Testing the reliability of the C-SCIB scale

The better the internal consistency of a scale, the better its reliability [22]. The overall Cronbach’s α of the 33 items in the C-SCIB scale was 0.81; the Cronbach’s α values of the internal locus of control, external locus of control, and support dimensions were 0.75, 0.71., and 0.88, respectively. The test-retest reliability showed that the overall scale Pearson’s correlation coefficient of the scale was 0.96; the Pearson’s correlation coefficient values of the internal locus of control, external locus of control, and support dimensions were 0.76, 0.78, and 0.97, respectively.

### Content validity index

The expert validity of the C-SCIB scale was tested in this study as well. The item-level content validity index (I-CVI) was 0.99; the scale-level content validity index (S-CVI) as evaluated by four experts with regard to content suitability was 0.96, 1, 1, and 1, respectively; the S-CVI with regard to clarity of text was 1, 0.96, 1, and 1, respectively, with a mean of 0.99. The S-CVI/UA (universal agreement) with regard to content suitability and clarity was 0.97 and 0.97, respectively. The I-CVI (0.99) and S-CVI (0.99) were both above 0.78 and 0.80. In general, an I-CVI greater than 0.78 and a S-CVI greater than 0.9 suggests good content validity and excellent expert validity [23].

### Criterion-related validity

The Questionnaire Measuring Attitudes About Labor and Delivery (QMAALD) developed by Marut and Mercer [24] was used as a criterion to measure the predictive validity of the C-SCIB scale. Pearson’s correlation coefficient was used to test the correlation between the QMAALD and the C-SCIB scale. The results showed a significant correlation between the two scales (*r* = 0.31, *p* < 0.01). All the dimensions in the C-SCIB scale had significant correlations with those in the QMAALD, such as the internal locus of control *(r* = 0.15, *p* < 0.05), the external locus of control (*r* = 0.30, *p* < 0.01), and the support dimension (*r* = .021, *p* < 0.01).

The Bryanton Adaptation of the Nursing Support in Labor Questionnaire (BANSILQ) developed by Bryanton, Fraser-Davry, and Sullian [25] and the Labor Agentry Scale (LAS) developed by Hodnett and Simmons-Tropea [26] were used as criteria, so as to analyze their correlations with all dimensions of the C-SCIB. The results showed a significant correlation between the BANSILQ and the support dimension of the C-SCIB scale (*r* = 0.49, *p* < 0.01). A significant correlation was also observed between the LAS and, respectively, the internal locus of control (*r* = 0.44, *p* < 0.01) and the external locus of control dimensions (*r* = 0.30, *p* < 0.01).

### Construct validity of the C-SCIB scale

The construct validity of the C-SCIB scale was evaluated through confirmatory factor analysis. The LISREL (8.54) software was used to assess the fit of the C-SCIB scale, with the 228 postpartum women as the parametric estimates. The basic assumptions of the structural equation model must be tested prior to analysis [27].

The assumptions were as follows: (1) No system missing values as the data of samples with missing values have been omitted; (2) An adequate sample size. There were 228 samples in this study, which exceeds the minimum sample size of 200; (3) The observed variables must be continuous and must have a minimum of four values while the data must be distributed normally. A five-point Likert scale was used in this study for evaluating the continuous variables. (4) Undesired parameter values must be checked [27].

Prior to confirmatory factor analysis, the sample data was checked for a normal distribution. The skewness coefficient and kurtosis coefficient should be approximately zero in a normal distribution, which suggests that the coefficients are not statistically significant. However, if the coefficients attain a significance level of 0.05, then they are significant and not equal to zero [28]. Kline suggested that the distribution of variables in a sample is not normal if the skewness coefficient and kurtosis coefficient of a variable is greater than 3 and 8, respectively. An extreme deviation from the normal distribution is possible when the kurtosis coefficient is greater than 20 [27]. The skewness coefficients and kurtosis coefficients of the sample data in this study ranged from − 0.005 to 0.750 and − 0.008 to 2.397, respectively. Hence, the distribution is considered to be normal.

**Fig. 2:**
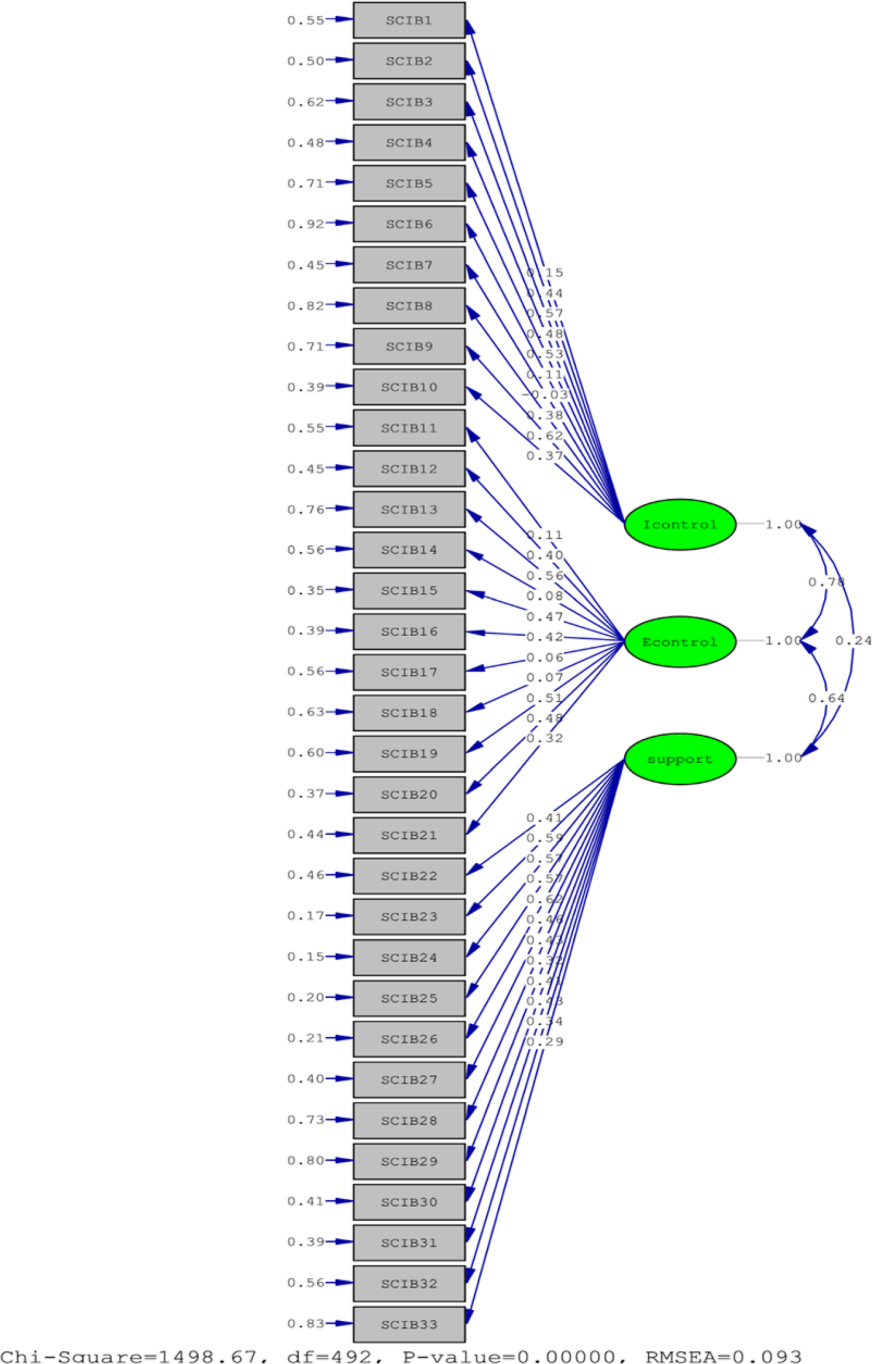
Flow diagram of the C-SCIB scale with three dimensions

With regard to the theoretical structure of the C-SCIB scale, three factors that consist of 33 items were developed in the preliminary measurement model (Fig. 2), so as to observe the parameter estimates and identify the model. The maximum likelihood estimation approach was used to estimate the model parameters and to evaluate the fit indexes of the overall C-SCIB scale. The results are shown in Table 3.

**Table 3:** The fit indexes of the overall C-SCIB scale (3 factor, 33 items)

	Results	Threshold	Judgment of fit
**Absolute fit measure indices**			
χ2 test	1498.27	*p* > .05	poor
RMSEA	.093	< .05	poor
GFI	.85	> .90	marginal
AGFI	.737	> .90	poor
**Incremental fit indices**			
IFI	.85	> .90	marginal
CFI	.85	> .90	marginal
NFI	.79	> .90	poor
TLI (NNFI)	.84	> .90	marginal
**Parsimony fit indices**			
PGFI	.64	> .50	well
PNFI	.50	> .50	well
PCFI	.588	> .50	well
χ2/df	3.105	< 2.00	poor

## Discussion

### Validity of the C-SCIB scale

In terms of expert validity, the I-CVI was 0.99 while the S-CVI as evaluated by four experts with regard to content suitability was 0.96, 1, 1, and 1, respectively; the S-CVI with regard to clarity of text was 1, 0.96, 1, and 1, respectively, with a mean of 0.99. The S-CVI/UA (universal agreement) was obtained by dividing the number of items in which a score of 4 of above was given by each expert with the number of total items (33). The results showed that the S-CVI/UA with regard to content suitability and clarity was 0.97 and 0.97, respectively. The I-CVI (0.99) and S-CVI (0.99) were both above 0.78 and 0.80. The expert validity of the scale is excellent as the experts gave a score of 4 or 5 for most items.

### Criterion-related validity

In this study, the QMAALD was used as a criterion and compared with the C-SCIB scale. The results revealed a significant but low correlation between the two scales. The dimensions of the C-SCIB scale, namely internal control, external control, emotional support, and tangible support, had a significant but low correlation with the QMAALD. This shows that the dimensions of the C-SCIB scale are correlated to the QMAALD, and even though the level of correlation is not high, the C-SCIB scale still exhibits some degree of predictive validity for QMAALD scores.

The BANSILQ and LAS were used as criteria and compared with the C-SCIB scale. The results revealed a significant and moderately positive correlation between the BANSILQ and the dimensions of emotional support and tangible support (*p* < 0.01). As for the LAS, it was found to be significantly correlated to the dimensions of internal control and external control (*p* < 0.01), with the correlation coefficients indicating a moderate correlation with internal control, a low correlation with external control, and a moderate correlation with the combined scores for internal and external control. This shows that C-SCIB scale possesses good concurrent validity.

Since the English language version of the scale was not subjected to a criterion-related validity test in previous studies, no comparisons were conducted. However, its subscales were compared, and the results indicated a high degree of correlation among its dimensions, with internal control and external control being moderately correlated (*r* = 0.55), internal control and support being moderately correlated (*r* = 0.51), and support and external control being highly correlated (*r* = 0.69). In our study, the correlations between the subscales were as follows: internal control and external control were found to be weakly correlated (*r* = 0.36), internal control and support were found to be moderately correlated (*r* = 0.46), and support and external control were found to be moderately correlated (*r* = 0.55). Despite the comparatively high degree of correlation among the subscales of the English-language scale, a significant correlation was still achieved in our study, indicating a link between control and support.

### Construct validity

In this study, a confirmatory factor analysis was performed to examine the construct validity of the SCIB scale when applied to postpartum women in Taiwan. An item analysis was first carried out before conducting the abovementioned analysis and five items were found to have not met the verification standards, namely 1. The labor pains were too much for me to gain control; 6. I felt my body was experiencing an event that was out of my control; 17. I was unable to control the staff entering and leaving the delivery room; 29. I felt the medical staff were only doing things for their own convenience; and 33. The medical staff ignored the things I said. The confirmatory factor analysis was eventually conducted with three factors, 33 items, and a sample size of 228 participants, with the aim of verifying the overall model’s goodness of fit. Although the results indicated a poor overall goodness of fit, they met the standards for parsimony goodness of fit. The researchers removed five items, reducing the total number of items from 33 to 28, and conducted another round of confirmatory analysis. Although the results were better compared to those for the initial 33 items, they still failed the standards for overall goodness of fit while meeting those for parsimony goodness of fit. Given that the confirmatory analysis results did not improve after several items were removed, the researchers decided to retain the original 33-item scale. In the original study, an exploratory factor analysis was performed, revealing factor loadings that were higher than 0.47 and a total variance explained of 55.8%. The original study primarily utilized statistical methods to identify common attributes shared by numerous variables, with the aim of establishing new hypotheses or developing new theoretical frameworks. In contrast, an exploratory factor analysis was not performed in our study, which only utilized confirmatory factor analysis to test and validate the scale model.

The above results indicate that the C-SCIB scale had good criterion-related validity and was able to effectively and quickly assess the support- and control-related conditions of women during childbirth, such that their birth experiences could be predicted. Despite the poor overall goodness of fit for construct validity, the results still met the standards required for parsimony goodness of fit and, thus, possess value as a reference.

The original English-language SCIB scale had an overall Cronbach’α coefficient of 0.95, and a coefficient of 0.86, 0.93, and 0.93 for internal control, external control, and support, respectively. In our study, the 33-item C-SCIB scale had an overall Cronbach’α coefficient of 0.81; a coefficient of 0.75, 0.71, and 0.88 for internal control, external control, and support, respectively; and a reliability in excess of 0.07. Even though these figures are lower compared to those in the original study, they nevertheless indicate a high degree of reliability. Furthermore, the scale’s test-retest reliability (tested using the Pearson product-moment correlation coefficient) was found to be 0.96 for the overall scale, and 0.76, 0.78, and 0.99 for internal control, external control, and support, respectively, indicating the scale’s stability. Since the English-language SCIB scale’s test-retest reliability was not tested, no comparisons between the two scales were performed. However, the C-SCIB scale’s reliability was higher than 0.70, which indicates that it has good test-retest reliability and stability.

In summary, the C-SCIB scale was found to have good reliability and stable test-retest reliability, and be correlated to the validity scales with respect to predictive and concurrent validity. Although the confirmatory factor analysis showed that overall goodness of fit was only for construct validity, its results still met the standards for parsimony goodness of fit. Like the original English-language scale, the C-SCIB scale was shown to have met the statistical standards for reliability and validity.

## Conclusion

The C-SCIB scale is a scale that combines support and control dimensions, and has been demonstrated to have considerably good reliability and validity, making it suitable for use in clinical and research settings. In a clinical setting, it can be used to assess the control and nursing care support provided to women during childbirth, such that we can predict whether their birth experiences will be positive or not. It is hoped that this study can serve as a reference for clinical nurses to perform evaluation and measurements, and be applied to research relating to birth satisfaction or experiences.

## Supplementary information

**Additional file 1. **English Support and Control in Birth scale (SCIB). The Support and Control in Birth scale (SCIB). The original (English) SCIB scale.

## Data Availability

All data generated or analyzed during this study are included in this published article.

## Abbreviations

SCIB: Support and Control in Birth
CSCIB: Chinese support and control in Birth
CVI: Content Validity Index
I-CVI: Item-level Content Validity Index
S-CVI: Scale-level Content Validity Index
LAS: Labor Agentry Scale
BANSILQ: The Bryanton Adaptation of the Nursing Support in Labor Questionnaire
QMAALD: Questionnaire Measuring Attitudes About Labor and Delivery Experience
