# Supplementary material for: Psychometric testing of the support and control in birth scale
Source: BMC Pregnancy Childbirth. 2020 May 14;20:293. doi: 10.1186/s12884-020-02888-x (PMC7222319; doi:10.1186/s12884-020-02888-x)
Supplement: Supplementary file 1 — Additional file 1. English Support and Control in Birth scale (SCIB). The Support and Control in Birth scale (SCIB). The original (English) SCIB scale. [file 12884_2020_2888_MOESM1_ESM.docx]

| Appendix: The Support and Control in Birth scale(SCIB) | | | | |  |  |
| --- | --- | --- | --- | --- | --- | --- |
| item | Strongly disagree | Disagree | Neutral | Agree | | Strongly agree |
| Internal control |  |  |  |  | |  |
| 1. The pain was too great for me to gain control over it ^a^ | 1 | 2 | 3 | 4 | | 5 |
| 2. I was overcome by the pain ^a^ | 1 | 2 | 3 | 4 | | 5 |
| 3. I was able to control my reactions to the pain | 1 | 2 | 3 | 4 | | 5 |
| 4. I was mentally calm | 1 | 2 | 3 | 4 | | 5 |
| 5. I was in control of my emotions | 1 | 2 | 3 | 4 | | 5 |
| 6. I felt my body was on a mission that I could not control ^a^ | 1 | 2 | 3 | 4 | | 5 |
| 7. Negative feelings overwhelmed me ^a^ | 1 | 2 | 3 | 4 | | 5 |
| 8. I gained control by working with my body | 1 | 2 | 3 | 4 | | 5 |
| 9. I could control the sounds I was making | 1 | 2 | 3 | 4 | | 5 |
| 10. I behaved in a way not like myself ^a^ | 1 | 2 | 3 | 4 | | 5 |
| External control |  |  |  |  | |  |
| 11. I had control over when procedures happened | 1 | 2 | 3 | 4 | | 5 |
| 12. I could influence which procedures were carried out | 1 | 2 | 3 | 4 | | 5 |
| 13. I decided whether procedures were carried out or not | 1 | 2 | 3 | 4 | | 5 |
| 14. The people in the room took control ^a^ | 1 | 2 | 3 | 4 | | 5 |
| 15. I had control over the decisions that were made | 1 | 2 | 3 | 4 | | 5 |
| 16. I could get up and move around as much as I wanted | 1 | 2 | 3 | 4 | | 5 |
| 17. People coming in and out of the room was beyond my control ^a^ | 1 | 2 | 3 | 4 | | 5 |
| 18. I chose whether I was given information or not | 1 | 2 | 3 | 4 | | 5 |
| 19. I could decide when I received information | 1 | 2 | 3 | 4 | | 5 |
| 20. I had control over what information I was given | 1 | 2 | 3 | 4 | | 5 |
| 21. I felt I had control over the way my baby was finally born | 1 | 2 | 3 | 4 | | 5 |
| Support |  |  |  |  | |  |
| 22. The staff helped me find energy to continue when I wanted to give up | 1 | 2 | 3 | 4 | | 5 |
| 23. The staff seemed to know instinctively what I wanted or needed | 1 | 2 | 3 | 4 | | 5 |
| 24. The staff went out of their way to try to keep me comfortable | 1 | 2 | 3 | 4 | | 5 |
| 25. The staff encouraged me to try new ways of coping (such as breathing) | 1 | 2 | 3 | 4 | | 5 |
| 26. The staff realized the pain I was in | 1 | 2 | 3 | 4 | | 5 |
| 27. The staff encouraged me not to fight against what my body was doing | 1 | 2 | 3 | 4 | | 5 |
| 28. I felt the staff had their own agenda ^a^ | 1 | 2 | 3 | 4 | | 5 |
| 29. I felt like the staff tried to move things along for their own convenience ^a^ | 1 | 2 | 3 | 4 | | 5 |
| 30. I was given time to ask questions | 1 | 2 | 3 | 4 | | 5 |
| 31. The staff helped me to try different positions | 1 | 2 | 3 | 4 | | 5 |
| 32. The staff stopped doing something if I asked them to stop | 1 | 2 | 3 | 4 | | 5 |
| 33. The staff dismissed things I said to them ^a^ | 1 | 2 | 3 | 4 | | 5 |
| Items with an "a" subscript were reversely scored | | | | |  |  |
